# Supplementary material for: Methionine consumption by cancer cells drives a progressive upregulation of PD-1 expression in CD4 T cells
Source: Nat Commun. 2023 May 5;14:2593. doi: 10.1038/s41467-023-38316-9 (PMC10162977; doi:10.1038/s41467-023-38316-9)
Supplement: Supplementary file 1 — Supplementary Information [file 41467_2023_38316_MOESM1_ESM.pdf]

# Supplementary Fig. 1

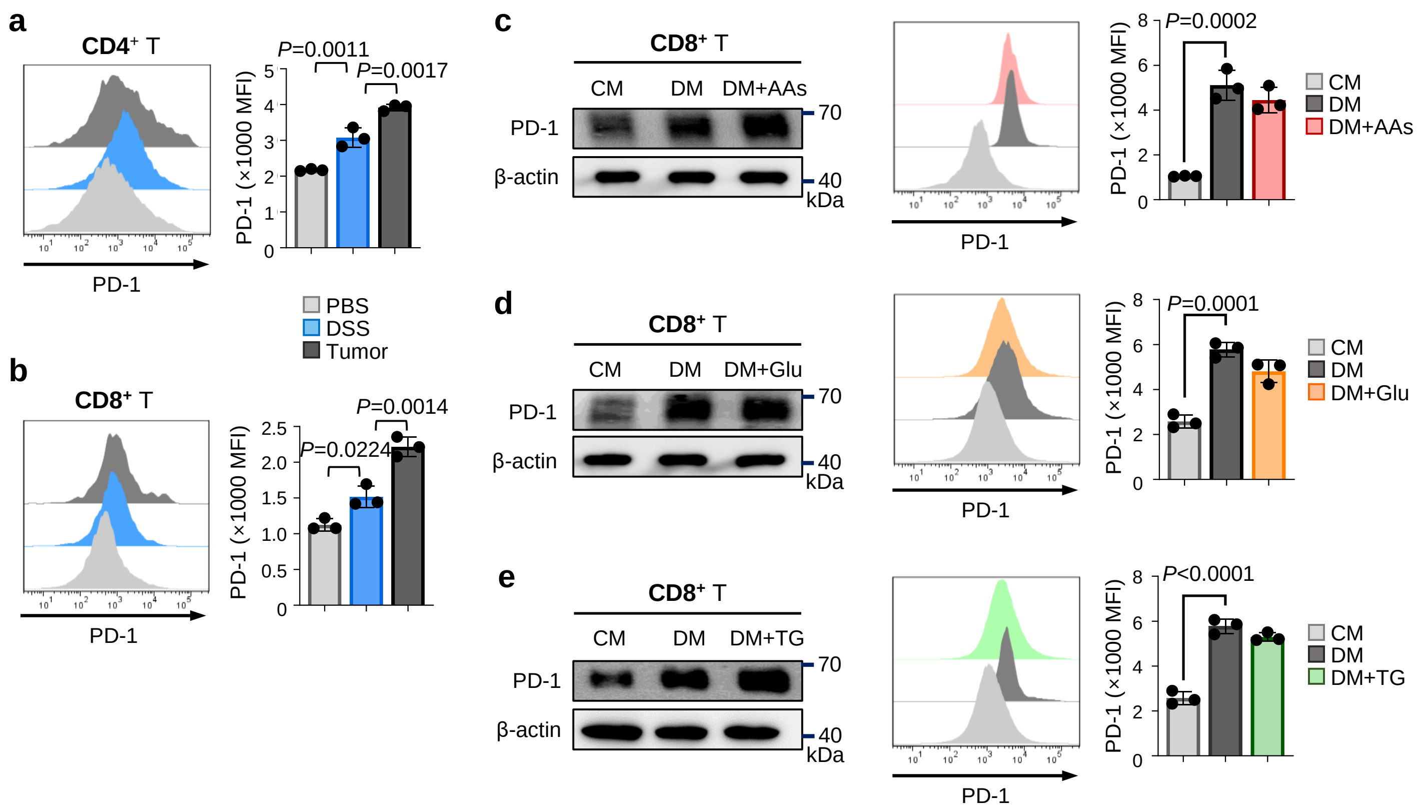

**Supplementary Fig. 1. Nutrient supplementation does not regulate PD-1 expression on CD8 T cells.**

**a–b.** CD4 and CD8 T cells were isolated from the lymph nodes of naïve, dextran sulfate sodium-induced colitis, and tumor-bearing mice. The MFI of PD-1 expression on CD4 **(a)** and CD8 T cells **(b)** was detected using FACS and is represented in the bar graph (n = 3 independent biological samples). **c–e.** Isolated CD8 T cells were cultured in CM, DM, and DM supplemented with nutrients (essential amino acids [AAs], glucose [Glu], and triglycerides [TG]). **c–e.** Immunoblots and the MFI of PD-1 expression in CD8 T cells cultured in CM, DM, and DM supplemented with AAs **(c)**, Glu **(d),** and TG **(e)** (n = 3 per group). Statistical analyses were performed using one-way analysis of variance (**a–e**). Data are mean ± standard error of the mean. Source data are provided as a Source Data file.

# Supplementary Fig. 2

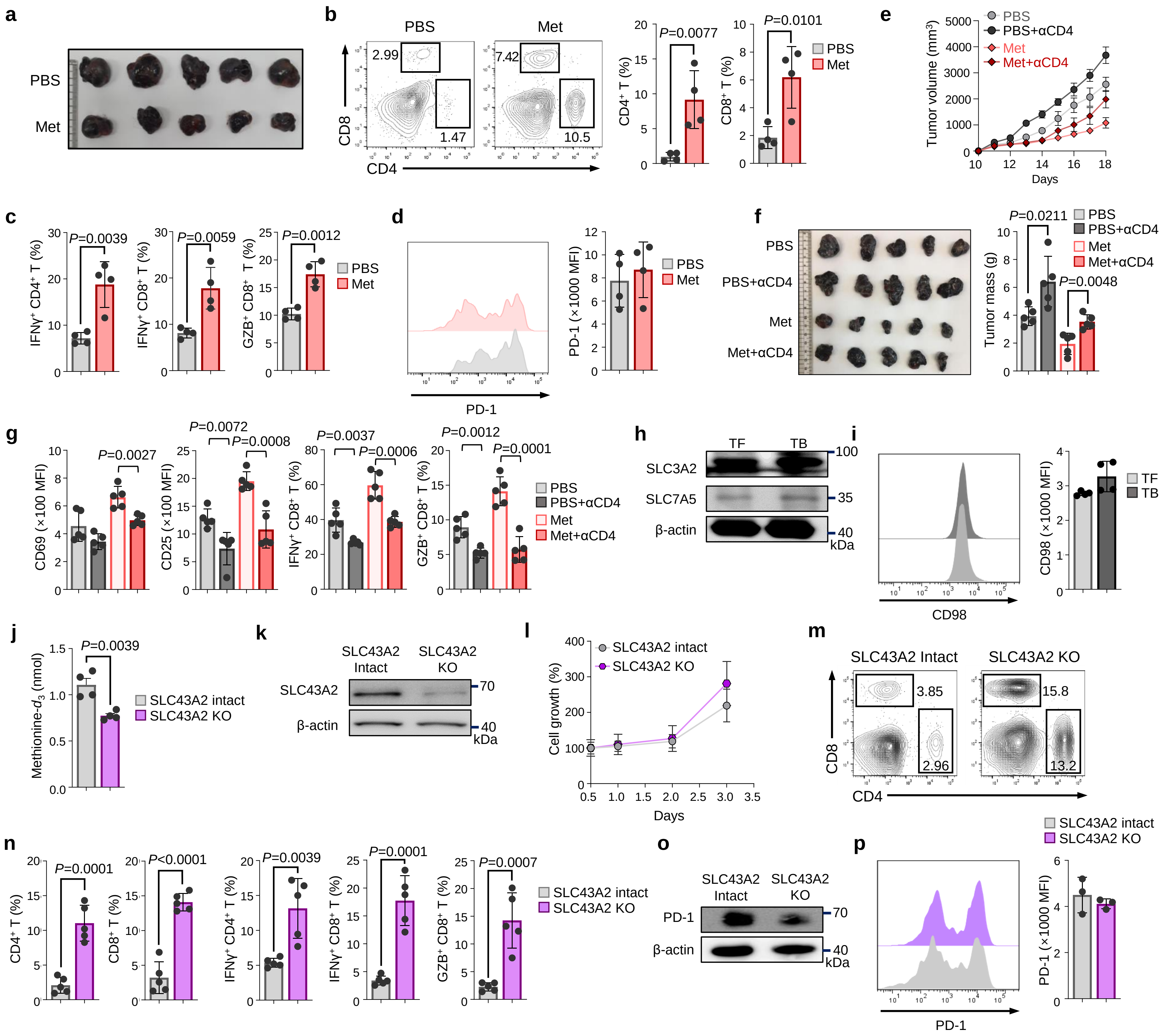

**Supplementary Fig. 2. Methionine confers antitumoral immunity.**

**a–d.** B16F10 tumor cells were injected into wild-type (WT) mice and PBS or methionine was administered intratumorally every other day starting from day 8 [n =5 (a) and n=4 (b-d) per group]. Tumor image **(a)**; percentage of tumor infiltrated CD4 and CD8 T cells **(b)**; IFN-γ and granzyme B (GZB) **(c)** and PD-1 expression in CD8 T cells **(d)**. **e–g.** B16F10 tumors transplanted in WT mice followed by treatment with methionine (40 mg/kg) and anti-CD4 antibodies (200 μg/i.p.), either alone or in combination (n = 5 per group). Tumor volume **(e)**, image and weight **(f)** and CD69 and CD25 expression and secretions of IFN-γ and GZB in tumor-infiltrated T cells **(g)**.

**h–i.** B16F10 tumor cells were transplanted into WT mice. Amino acid transporters SLC3A2 and SLC7A5 were detected by western blotting **(h)** and their heterodimer form CD98 by FACS (n = 4 per group) **(i)** in CD4 T cells isolated from LN of tumor-free mice and dLN of tumor-bearing mice. **j.** Uptake assay to analyze uptake of methionine by SLC43A2 intact and KO B16F10 cells by the addition of methionine-d3 (200μM) to culture medium for 1h and detected by LC/MS (n=4 per group). **k.** Immunoblots to confirm *SLC43A2* deletion using CRISPR-Cas9. The experiment was repeated two times. **l.** Comparison of the cell growth rate of SLC43A2-intact and KO B16F10 cells using MTT assay (n = 3 per group). **m–p.** SLC43A2 intact and KO B16F10 tumor cells were subcutaneously injected into WT mice (n = 5 per group). Percentages of tumor-infiltrated CD4 and CD8 T cells and IFN-γ and GZB secretions **(m–n)**. Immunoblotting of PD-1 in tumor-infiltrating CD4 T cells. The experiments were repeated two times ( **o).** PD-1 expression in tumor-infiltrating CD8 T cells (n = 3 per group) (**p**). Statistical analyses were performed using two-tailed Student's *t*-test **(b-d, i-j, n,p) ;** two-way ANOVA **(e, l).** PBS vs PBS + αCD4 $P$<0.0001; Met vs Met + αCD4 $P$<0.0001 **(e)**; one-way ANOVA **(f–g)**. Data are mean ± standard error of the mean. Source data are provided as a Source Data file.

# Supplementary Fig. 3

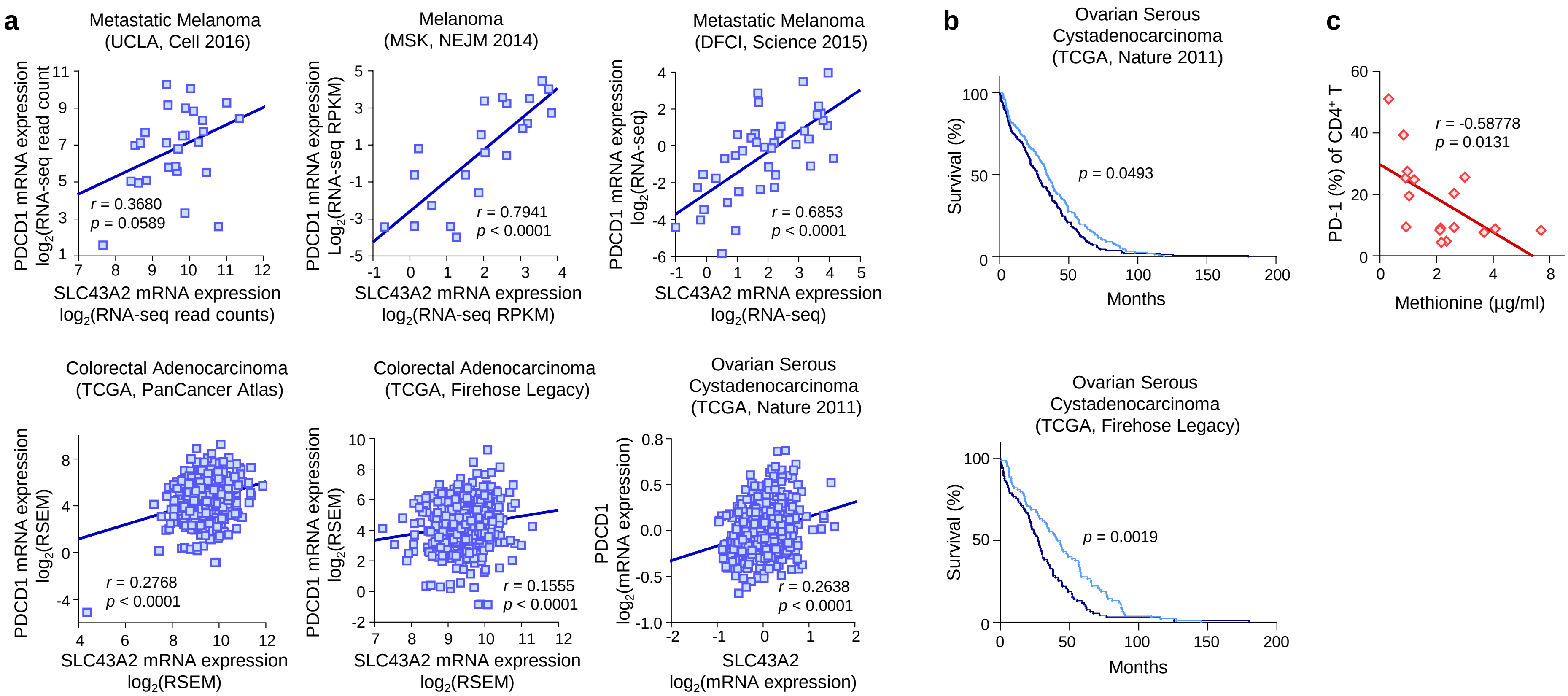

**Supplementary Fig. 3. Positive correlation between CD4 T cells, PD-1, and *SLC43A2* mRNA expression in multiple cancer patient datasets. A.** Transcriptional correlation between transcripts of SLC43A2 and CD4 T cell PDCD1 analyzed from The Cancer Genome Atlas dataset. Metastatic Melanoma[1] (UCLA, Cell 2016, N=27), Melanoma[2] (MSK, NEJM 2014, N=21), Metastatic Melanoma[3] (DFCI, Science 2015, N=40), Colorectal Adenocarcinoma[4-13] (TCGA, PanCancer Atlas, N=592), Colorectal Adenocarcinoma (TCGA, Firehose Legacy, N=382), Ovarian Serous Cystadenocarcinoma[14] (TCGA, Nature 2011, N=489) **b.** Survival graph of cancer patients with high and low SLC43A2 expression. Median survival was assessed using the Kaplan–Meier curve. Ovarian Serous Cystadenocarcinoma[14] (TCGA, Nature 2011, SLC43A2 low N=157, SLC43A2 high N=158), Ovarian Serous Cystadenocarcinoma (TCGA, Firehose Legacy, SLC43A2 low N=90, SLC43A2 high N=91) **c.** Correlation between PD-1 expression in CD4 T cells and methionine concentration. Statistical analyses were performed using Pearson correlation analysis **(a, c)** and Log-rank test **(b).** Source data are provided as a Source Data file.

# Supplementary Fig. 4

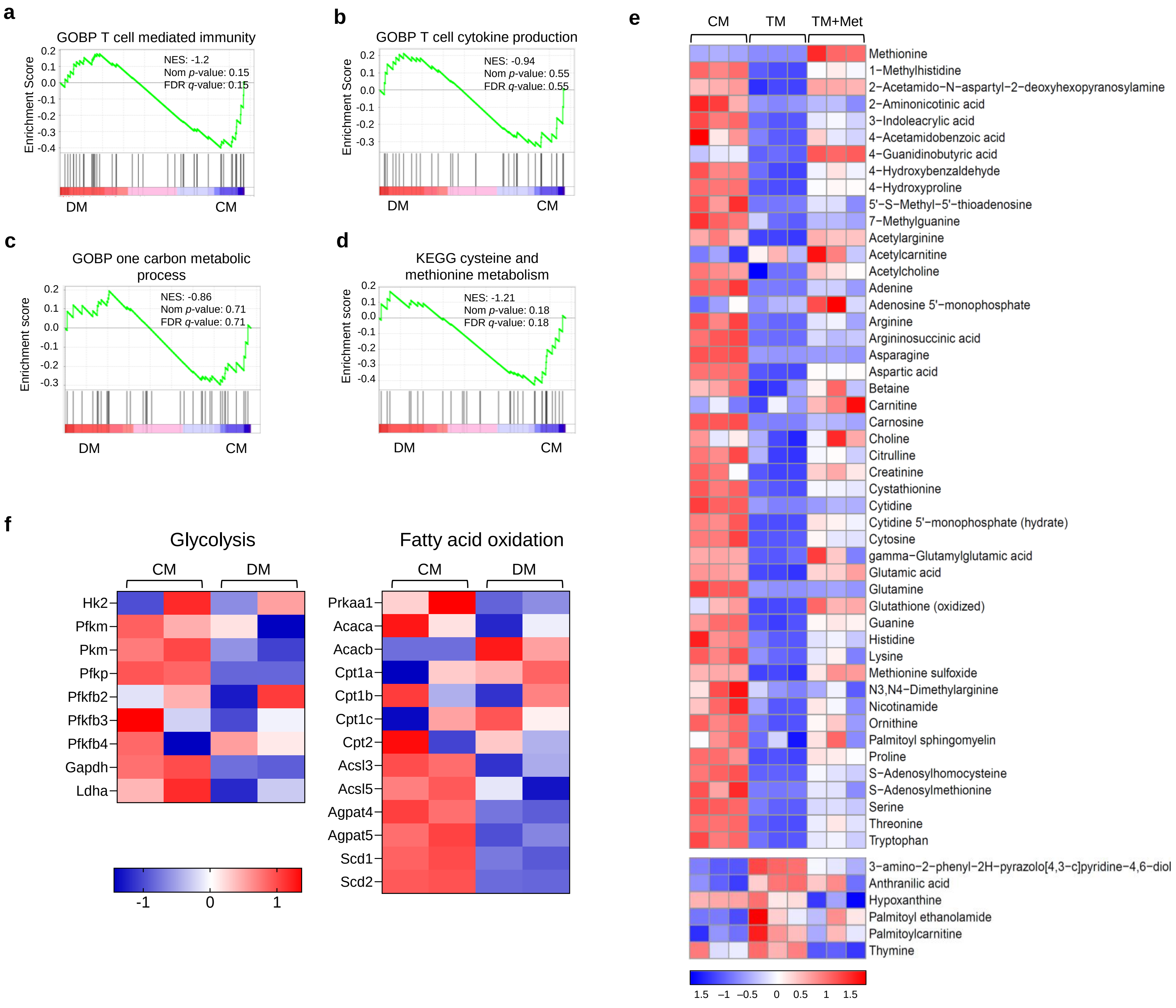

**Supplementary Fig. 4. CD4 T cells cultured in reduced-nutrient media exhibit reduced levels of amino acids, metabolites, and metabolism-related genes. a–d.** GSEA of CD4 T cells cultured in CM and DM (n = 2 independent biological samples). T cell-mediated immunity pathways **(a)**, T cell cytokine production **(b)**, one-carbon metabolic process **(c),** and KEGG cysteine and methionine metabolism **(d)** in CD4 T cells. **e**. Metabolomic analysis of CD4 T cells cultured in CM, TM, and TM supplemented with methionine (n = 3 independent biological samples). **f.** RNA-seq analysis of glycolysis(left) and fatty acid oxidation (right)-related gene expression in CD4 T cells cultured in CM and TM (n = 2 independent biological samples). Statistical analyses were performed using two-tailed Student's *t*-test & TMM+CPM normalized method for correction **(a-d).** Source data are provided as a Source Data file.

# Supplementary Fig. 5

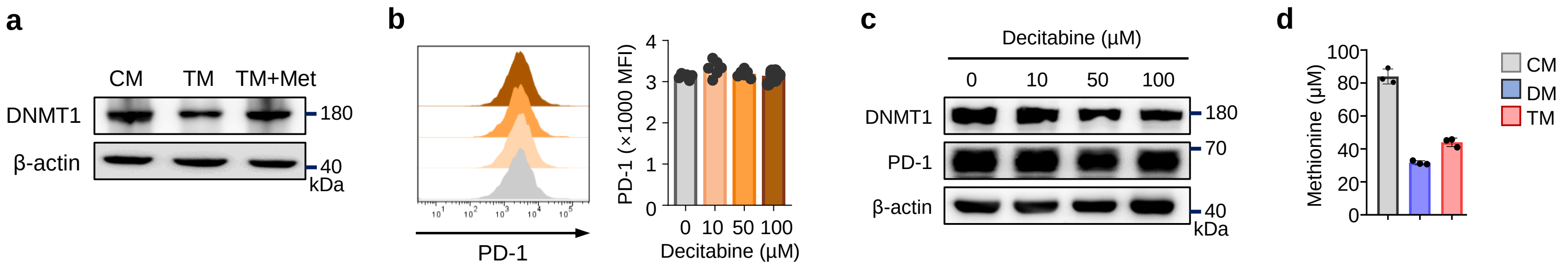

**Supplementary Fig. 5. DNA methylation does not regulate PD-1 expression.**

**a.** Immunoblot for the DNMT1 analysis in CD4 T cells cultured in CM, TM, or TM supplemented with methionine. The experiments were performed twice with similar results. **b–c.** The DNMT inhibitor decitabine was administered at different dosages. PD-1 expression was analyzed using flow cytometry (n = 6 per group) **(b)**, and DNMT1 and PD-1 expression analyzed using western blotting **(c)**. Methionine level in CM, DM and TM (n=3 in each group) **(d)**. The experiments were repeated twice with similar results. One-way ANOVA was performed **(b)**. Data are mean ± standard error of the mean. Source data are provided as a Source Data file.

# Supplementary Fig. 6

**a**

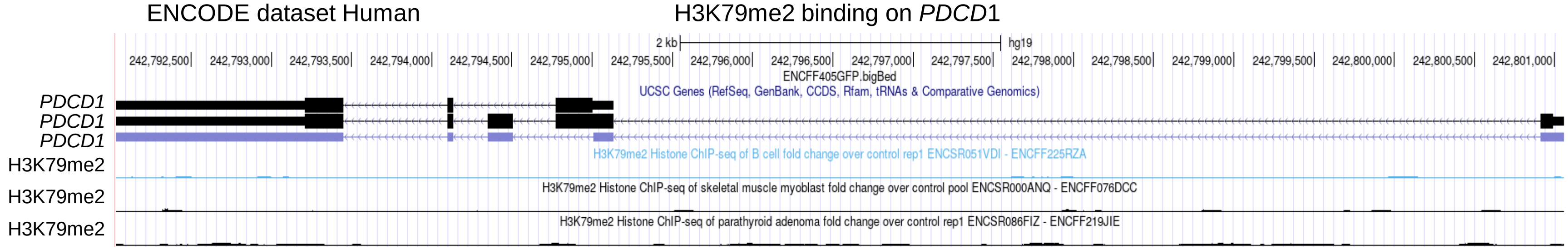


**b**

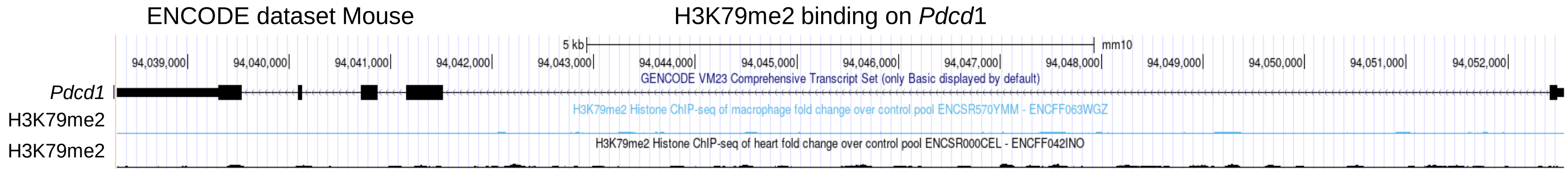


**c**

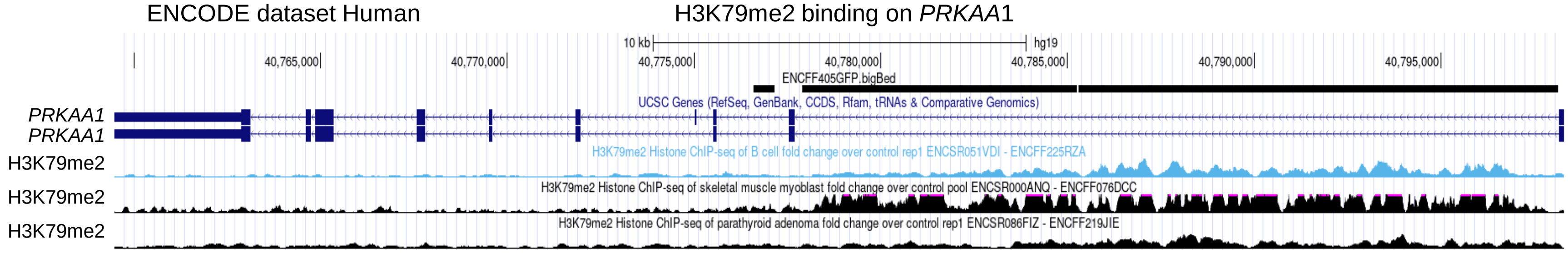


**d**

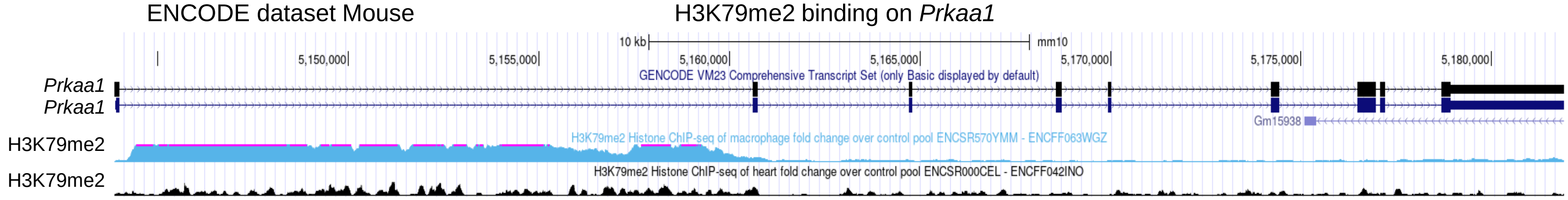


**e**

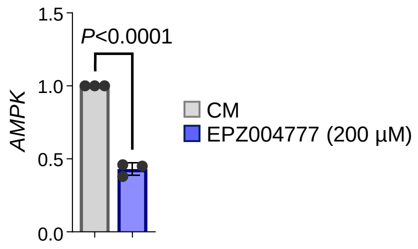

**Supplementary Fig. 6. H3K79me2 binds with the AMPK gene promoter.**

**a–b.** H3K79me2 ChIP-seq data in the ENCODE public dataset on different human and mouse cell types show the binding of H3K79me2 to the *Pdcd1* promoter. **c–d.** H3K79me2 ChIP-seq data in the ENCODE public dataset on different cell types of humans and mice show H3K79me2 binding in the *Prkaa1* promoter. **e.** Isolated CD4 T cells were cultured in the presence or absence of DOT1L inhibitor EPZ004777(200μM) for 12 h. RT PCR was performed for the detection of AMPKα1 mRNA (n=3 per group). Statistical analyses were performed using two-tailed Student's *t*-test **(e)**. Data are mean ± standard error of the mean. Source data are provided as a Source Data file.

# Supplementary Fig. 7

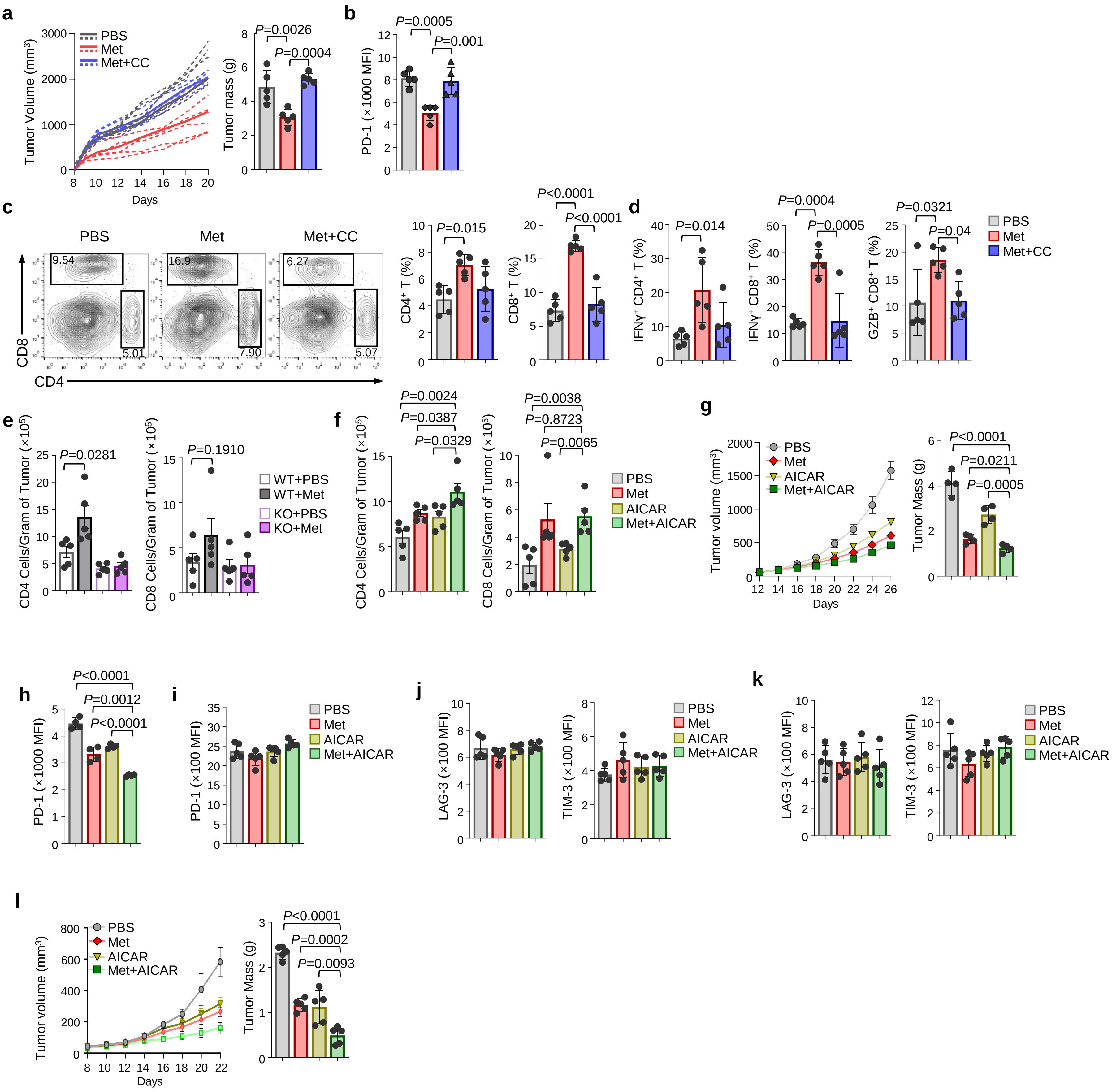

**Supplementary Fig. 7. Methionine exhibits antitumoral immunity via AMPK.**

**a–d.** B16F10 cells were subcutaneously injected into WT mice. Methionine (intratumoral) and compound C (intraperitoneal) were administered every other day starting on day 9 (n = 5 per group). **a.** Tumor volume and weight **b**. PD-1 expression on tumor-infiltrated CD4 T cells. **c.** Percentages of tumor-infiltrated CD4 and CD8 T cells. **d.** IFN-γ and GZB cytokine secretions by tumor-infiltrating T cells. **e.** Absolute cell counts of tumor infiltrating CD4 and CD8 T cells of WT and AMPK KO mice transplanted with B16F10 cells following treatment with methionine (40 mg/kg; intra-tumor) or PBS every alternate day starting on day 8 (n = 5 per group). **f**. Absolute cell count of tumor-infiltrating CD4 and CD8 T cells from mice transplanted with B16F10 cells followed by treatment with PBS or methionine (40 mg/kg) or AICAR (500 mg/kg) every alternate day starting on day 8 (n = 5 per group). **g-h.** WT mice were transplanted with MC-38 cells followed by treatment with PBS or methionine (40 mg/kg) or AICAR (500 mg/kg) every other day starting on day 12 (n = 4 per group). **g.** Tumor volume and weight **h.** PD-1 expression on tumor-infiltrated CD4 T cells. **i**. PD-1 expression on CD4 T cells derived from lymph nodes. **j.** LAG-3 and TIM-3 expression in CD4 T cells derived from lymph nodes of tumor-bearing mice (n=5 per group). **k.** LAG-3 and TIM-3 expression of tumor-infiltrating CD4 T cells (n=5 per group). **l.** WT mice were transplanted with SLC43A2-KO B16F10 cells followed by treatment with PBS or methionine (40 mg/kg) or AICAR (500 mg/kg) every other day starting on day 8 (n = 5 per group) and tumor volume and weight were analyzed. Tumor volume was analyzed using two-way ANOVA (PBS vs. Met $P<0.0001$; PBS vs. Met+CC $P=0.1970$) **(a),** PBS vs. Met+AICAR $P<0.0001$; Met vs Met+AICAR $P=0.0267$; AICAR vs. Met+AICAR $P<0.0001$) **(g)**; PBS vs. Met+AICAR $P<0.0001$; Met vs Met+AICAR $P<0.0001$; AICAR vs. Met+AICAR $P<0.0001$) **(l)**;one-way ANOVA multiple comparison tests were performed **(a–l)**. Data are mean ± standard error of the mean. Source data are provided as a Source Data file.

# Supplementary Fig. 8

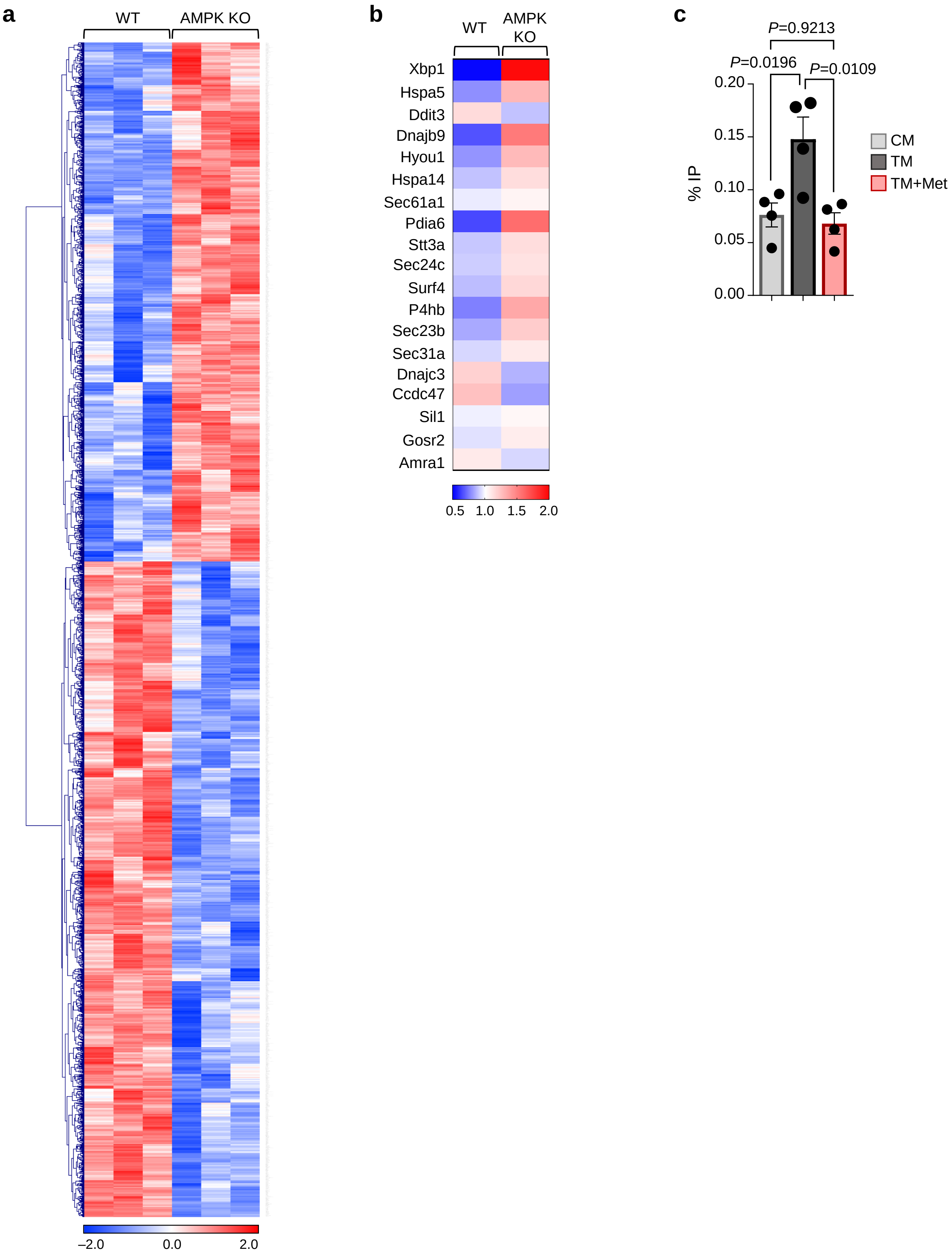

**Supplementary Fig. 8. AMPK-deficient CD4 T cells exhibit increased expression of endoplasmic reticulum (ER) stress-related genes. a.** RNA-seq analysis showing differentially expressed genes in AMPK-deficient CD4 T cells. **b.** RNA-seq analysis of ER stress-related gene expression in wild-type (WT) and AMPK-deficient CD4 T cells. (**a, b**; n = 3 biological independent samples). **c**. Chip assay showing XBP1 binding to PD-1 promoter (n=4 per group). Statistical analysis was performed using one-way ANOVA multiple comparison test (c). Data are mean ± standard error of the mean. Source data are provided as a Source Data file.

# Supplementary Fig. 9

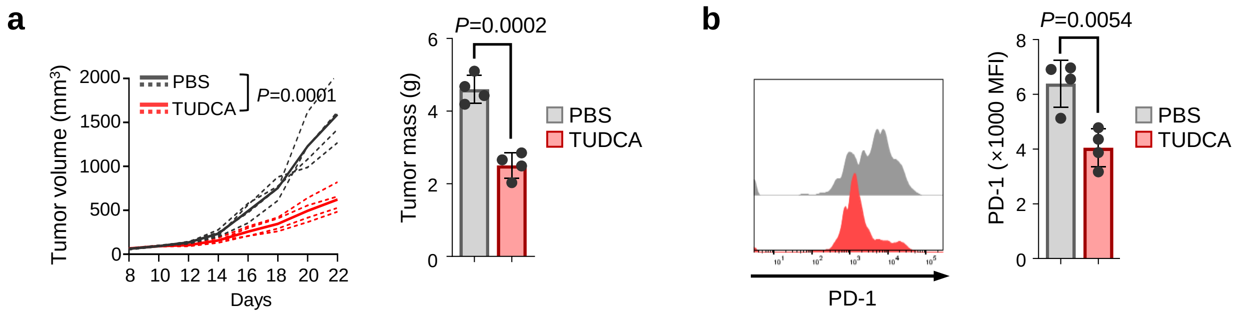

**Supplementary Fig. 9. ER stress inhibitor TUDCA exhibits anti-tumor immunity.**

**a-b.** WT mice transplanted with B16F10 melanoma cells and treated with TUDCA (150 mg/kg; intra-peritoneal) or control phosphate-buffered saline (PBS) every alternate day (n = 4 per group)**. a.** Tumor volume and tumor weight were analyzed. **b.** PD-1 expression of tumor-infiltrating CD4. Statistical analyses were performed using two-way analysis of variance (ANOVA) for tumor volume **(a)** and two-tailed student's *t*-test were performed for bar graphs **(a-b).** Data are mean ± standard error of the mean. Source data are provided as a Source Data file.

# Supplementary Fig. 10

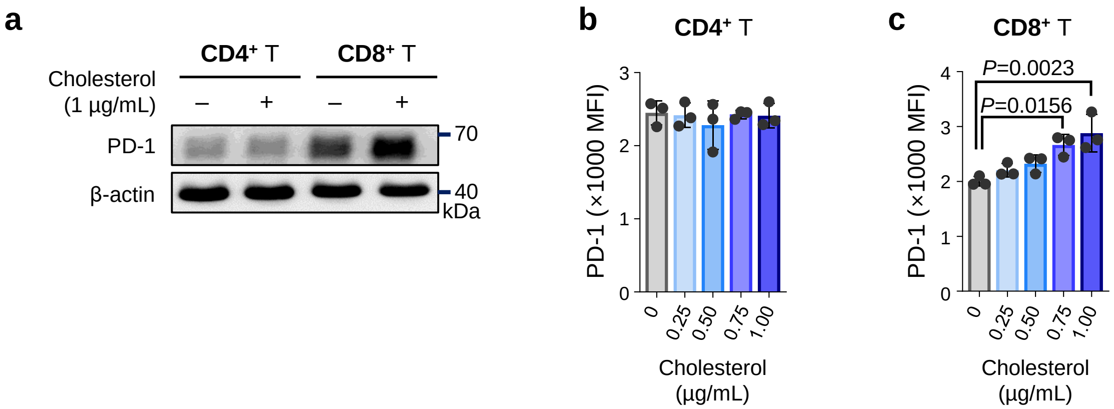

**Supplementary Fig. 10. Cholesterol supplementation does not regulate PD-1 expression on CD4 T cells. a**. Immunoblotting showing PD-1 on CD4 or CD8 T cells after treatment with cholesterol. The experiments were performed two times with similar results. **b.** Flow cytometry analysis of PD-1 in CD4 T cells treated with different doses of cholesterol. **c.** Flow cytometry analysis of PD-1 in CD8 T cells treated with different doses of cholesterol. (**b, c**; n = 3 per group). Statistical analyses were performed using one-way analysis of variance (**b,c**). Data are mean ± standard error of the mean. Source data are provided as a Source Data file.

# Supplementary Fig. 11

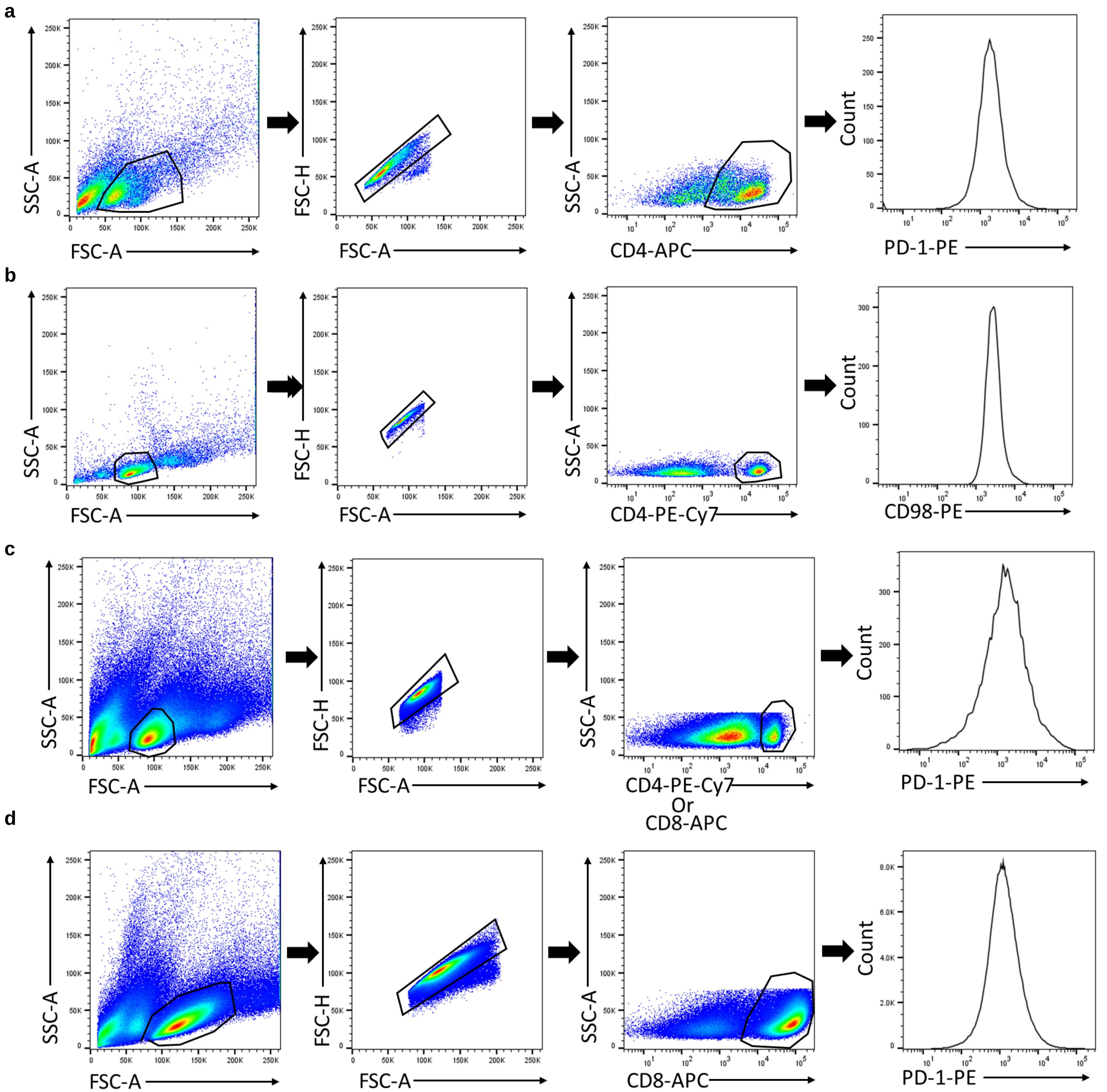

**Supplementary Fig. 11. Gating Strategy**

**a.** Gating strategy to find PD-1 expression on CD4 T cells (Used in figure 1d, 3j and Supplementary figure 5b and 10b. **b.** Gating strategy to find CD98 expression on CD4 T cells (Used in Supplementary figure 2i). **c.** Gating strategy to find PD-1 expression on CD4 T cells and CD8 T cells (Used in Supplementary figure 1a and 1b). **d.** Gating strategy to find PD-1 expression on CD8 T cells (Supplementary 1c, 1d, 1e and 10c).

# Supplementary Fig. 12

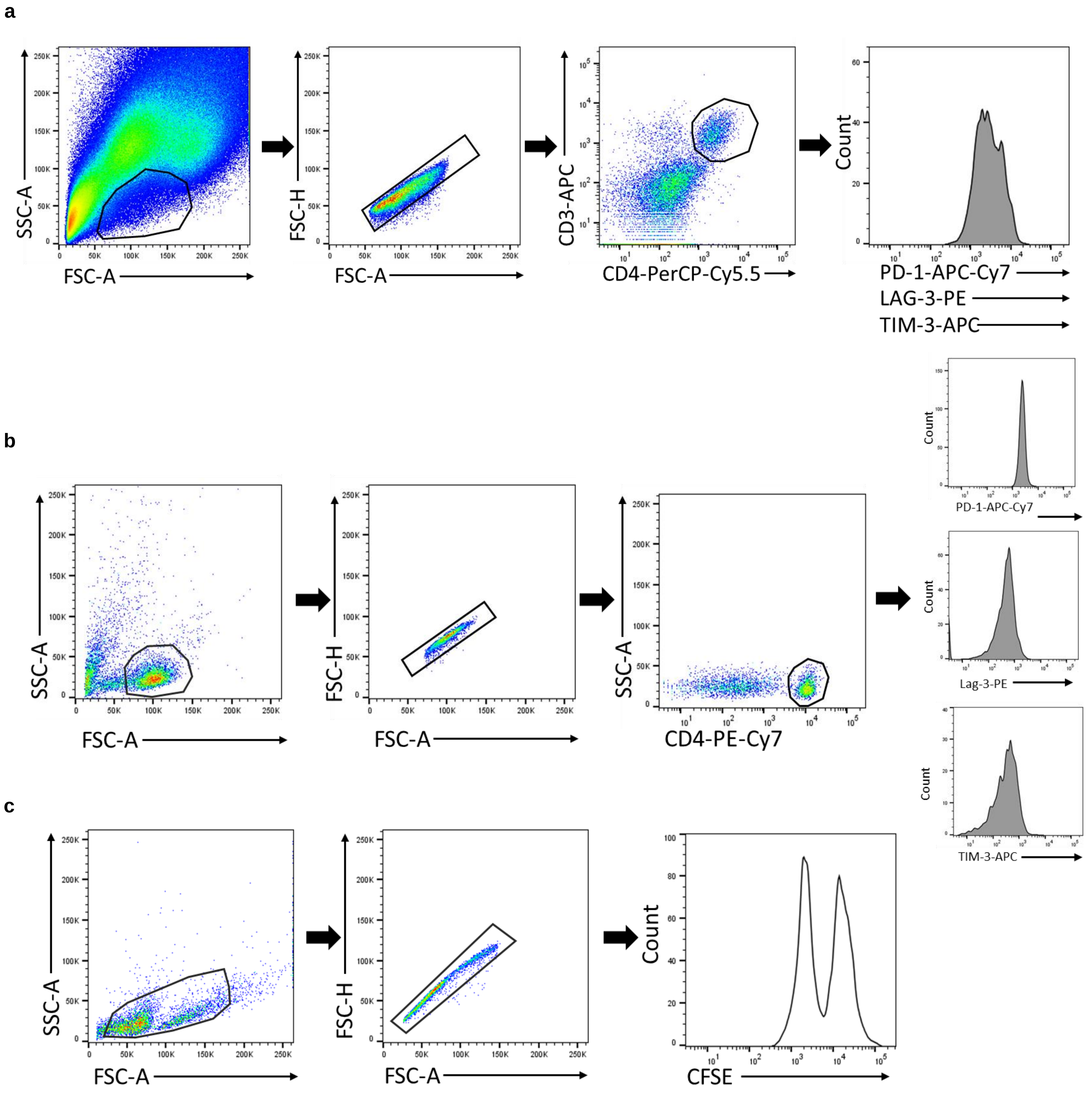

**Supplementary Fig. 12. Gating Strategy**

**a.** Gating strategy to find PD-1, LAG-3 and TIM-3 expression on tumor infiltrated CD4 T cells (Used in figure 2b, 2f, 5b, 5d, 5h and Supplementary figure 2d, 2p, 7b, 7h, 7k and 9b). **b.** Gating strategy to find PD-1, LAG-3 and TIM-3 expression on CD4 T cells derived from lymph nodes of tumor mice (Used in Supplementary figure 7i and 7j). **c.** Gating strategy to find low and high CFSE labelled cells in *in vitro* cytotoxicity assay (Used in figure 2d).

# Supplementary Fig. 13

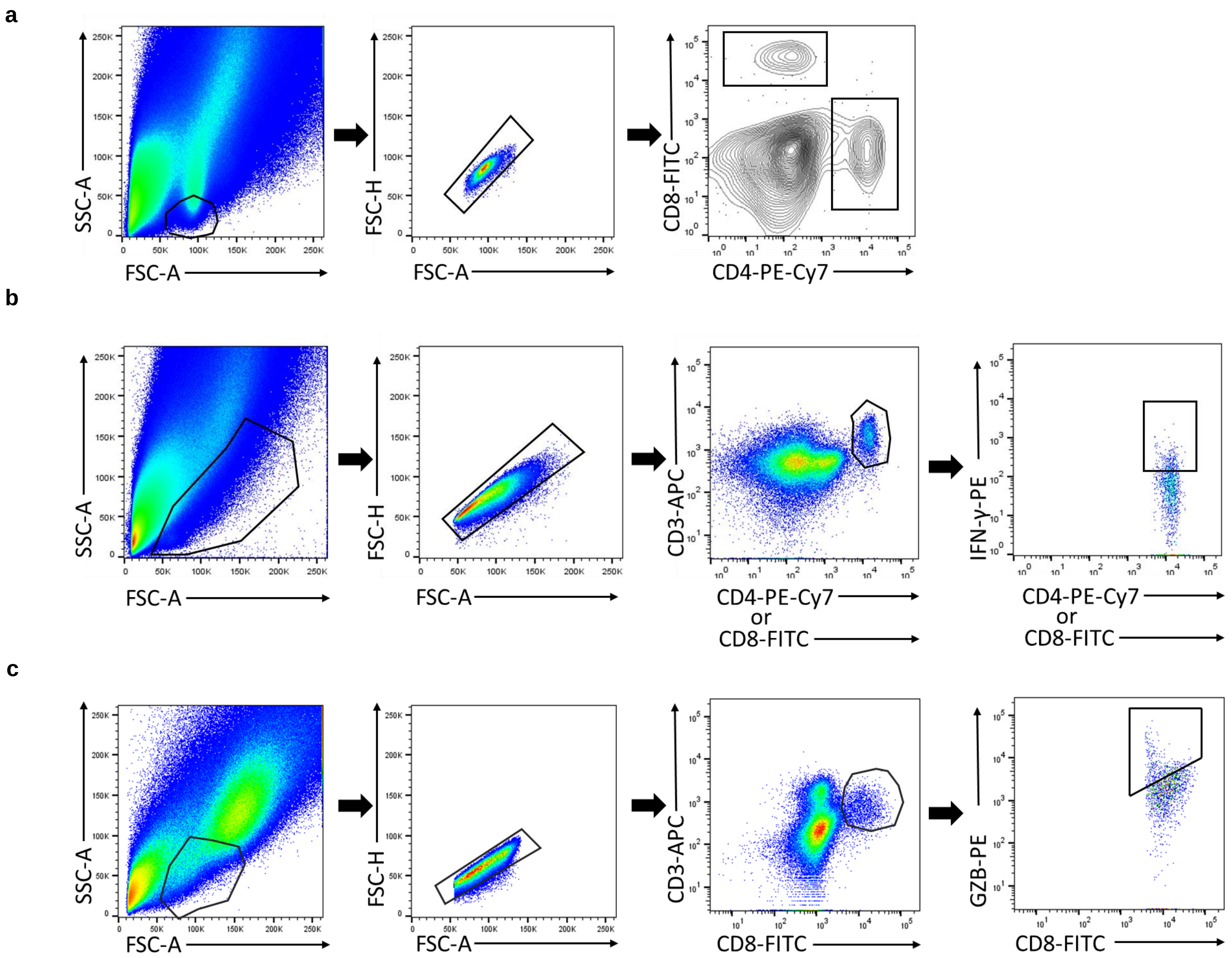

**Supplementary Fig. 13. Gating Strategy**

**a.** Gating strategy to identify tumor infiltrated CD4 T cells and CD8 T cells (Used in figure 5e, 5i and Supplementary figure 2b, 2m and 7c). **b.** Gating strategy to identify IFN-γ producing tumor infiltrated CD4 T cells and CD8 T cells (Used in figure 5f, 5j and Supplementary figure 2c, 2n and 7d). **c.** Gating strategy to identify GZB producing tumor infiltrated CD8 T cells (Used in figure 5f, 5j and Supplementary figure 2c, 2n and 7d).

# Supplementary Table 1

**Supplementary Table 1. Real time PCR primer list with sequences**

| **Genes** | **Forward (5′–3′)** | **Reverse (5′–3′)** |
|---|---|---|
| *Prkaa1* | GAAGTCAAAGCCGACCCAAT | AGGGTTCTTCCTTCGCACAC |
| *XBP1* | GACAGAGAGTCAAACTAACGTGG | GTCCAGCAGGCAAGAAGGT |
| *XBP1s* | AAGAACACGCTTGGGAATGG | CTGCACCTGCTGCGGAC |
| *Pdcd1* | ATGTGGGTCCGGCAGGTACC | TCAAAGAGGCCAAGAACAATGTC |

## Reference


1. Hugo, W. *et al.* Genomic and Transcriptomic Features of Response to Anti-PD-1 Therapy in Metastatic Melanoma. *Cell* 165, 35-44, doi:10.1016/j.cell.2016.02.065 (2016).

2. Snyder, A. et al. Genetic basis for clinical response to CTLA-4 blockade in melanoma. The New England journal of medicine 371, 2189-2199, doi:10.1056/NEJMoa1406498 (2014).

3. Van Allen, E. M. et al. Genomic correlates of response to CTLA-4 blockade in metastatic melanoma. Science 350, 207-211, doi:10.1126/science.aad0095 (2015).

4. Hoadley, K. A. *et al.* Cell-of-Origin Patterns Dominate the Molecular Classification of 10,000 Tumors from 33 Types of Cancer. *Cell* 173, 291-304.e296, doi:10.1016/j.cell.2018.03.022 (2018).

5. Ellrott, K. *et al.* Scalable Open Science Approach for Mutation Calling of Tumor Exomes Using Multiple Genomic Pipelines. *Cell systems* 6, 271-281.e277, doi:10.1016/j.cels.2018.03.002 (2018).

6. Taylor, A. M. *et al.* Genomic and Functional Approaches to Understanding Cancer Aneuploidy. *Cancer Cell* 33, 676-689.e673, doi:10.1016/j.ccell.2018.03.007 (2018).

7. Liu, J. *et al.* An Integrated TCGA Pan-Cancer Clinical Data Resource to Drive High-Quality Survival Outcome Analytics. *Cell* 173, 400-416.e411, doi:10.1016/j.cell.2018.02.052 (2018).

8. Sanchez-Vega, F. et al. Oncogenic Signaling Pathways in The Cancer Genome Atlas. Cell 173, 321-337.e310, doi:10.1016/j.cell.2018.03.035 (2018).

9. Gao, Q. *et al.* Driver Fusions and Their Implications in the Development and Treatment of Human Cancers. *Cell reports* 23, 227-238.e223, doi:10.1016/j.celrep.2018.03.050 (2018).

10. Bhandari, V. *et al.* Molecular landmarks of tumor hypoxia across cancer types. *Nature genetics* 51, 308-318, doi:10.1038/s41588-018-0318-2 (2019).

11. Poore, G. D. *et al.* Microbiome analyses of blood and tissues suggest cancer diagnostic approach. *Nature* 579, 567-574, doi:10.1038/s41586-020-2095-1 (2020).

12. Ding, L. *et al.* Perspective on Oncogenic Processes at the End of the Beginning of Cancer Genomics. *Cell* 173, 305-320.e310, doi:10.1016/j.cell.2018.03.033 (2018).

13. Bonneville, R. *et al.* Landscape of Microsatellite Instability Across 39 Cancer Types. *JCO precision oncology* 2017, doi:10.1200/po.17.00073 (2017).

14. Integrated genomic analyses of ovarian carcinoma. Nature 474, 609–615, doi:10.1038/nature10166 (2011).
