# Supplementary material for: Methionine consumption by cancer cells drives a progressive upregulation of PD-1 expression in CD4 T cells
Source: Nat Commun. 2023 May 5;14:2593. doi: 10.1038/s41467-023-38316-9 (PMC10162977; doi:10.1038/s41467-023-38316-9)
Supplement: Supplementary file 3 — Description of Additional Supplementary Files [file 41467_2023_38316_MOESM3_ESM.pdf]

Description of Additional Supplementary Files

Supplementary Data 1: Metabolomic analysis of CD4 T cells cultured in CM, TM, and TM supplemented with methionine representing changes in wide panel of primary metabolites (n = 3 per group). Metabolites were quantified by using Gas chromatography with quadrupole time-of-flight mass spectrometry (GC-TOF-MS) and the relative value of each metabolite between the groups were measured. Each value is normalized and expressed in Log2fold change.

Supplementary Data 2: Metabolomic analysis of CD4 T cells cultured in CM, TM, and TM supplemented with methionine representing changes in wide panel of primary metabolites (n = 3 per group). Metabolites were quantified by using Gas chromatography with quadrupole time-of-flight mass spectrometry (GC-TOF-MS). The raw values of each metabolites are expressed in column.
