# Supplementary material for: Methionine consumption by cancer cells drives a progressive upregulation of PD-1 expression in CD4 T cells
Source: Nat Commun. 2023 May 5;14:2593. doi: 10.1038/s41467-023-38316-9 (PMC10162977; doi:10.1038/s41467-023-38316-9)
Supplement: Supplementary file 6 — Reporting Summary [file 41467_2023_38316_MOESM6_ESM.pdf]

nature portfolio

Corresponding author(s): Jae-Hoon Chang

Last updated by author(s): Apr 21, 2023

Reporting Summary

Nature Portfolio wishes to improve the reproducibility of the work that we publish. This form provides structure for consistency and transparency in reporting. For further information on Nature Portfolio policies, see our Editorial Policies and the Editorial Policy Checklist.

Statistics

For all statistical analyses, confirm that the following items are present in the figure legend, table legend, main text, or Methods section.

n/a	Confirmed	
☐	☒	The exact sample size (*n*) for each experimental group/condition, given as a discrete number and unit of measurement
☐	☒	A statement on whether measurements were taken from distinct samples or whether the same sample was measured repeatedly
☐	☒	The statistical test(s) used AND whether they are one- or two-sided<br>*Only common tests should be described solely by name; describe more complex techniques in the Methods section.*
☐	☒	A description of all covariates tested
☐	☒	A description of any assumptions or corrections, such as tests of normality and adjustment for multiple comparisons
☐	☒	A full description of the statistical parameters including central tendency (e.g. means) or other basic estimates (e.g. regression coefficient) AND variation (e.g. standard deviation) or associated estimates of uncertainty (e.g. confidence intervals)
☐	☒	For null hypothesis testing, the test statistic (e.g. *F*, *t*, *r*) with confidence intervals, effect sizes, degrees of freedom and *P* value noted<br>*Give P values as exact values whenever suitable.*
☒	☐	For Bayesian analysis, information on the choice of priors and Markov chain Monte Carlo settings
☐	☒	For hierarchical and complex designs, identification of the appropriate level for tests and full reporting of outcomes
☒	☐	Estimates of effect sizes (e.g. Cohen's *d*, Pearson's *r*), indicating how they were calculated

Our web collection on statistics for biologists contains articles on many of the points above.

Software and code

Policy information about availability of computer code

Data collection	Flowcytometer BD FACSVerse was used to run samples. The data was analyzed by with FlowJo version10
Data analysis	GraphPad Prism version 8 and 9 were used for data analysis.

For manuscripts utilizing custom algorithms or software that are central to the research but not yet described in published literature, software must be made available to editors and reviewers. We strongly encourage code deposition in a community repository (e.g. GitHub). See the Nature Portfolio guidelines for submitting code & software for further information.

Data

Policy information about availability of data

All manuscripts must include a data availability statement. This statement should provide the following information, where applicable:

- Accession codes, unique identifiers, or web links for publicly available datasets
- A description of any restrictions on data availability
- For clinical datasets or third party data, please ensure that the statement adheres to our policy

The RNAseq data generated in this study are available in the GEO database under accession code GSE210182 (https://www.ncbi.nlm.nih.gov/geo/query/acc.cgi?acc=GSE210182) and GSE210183 (https://www.ncbi.nlm.nih.gov/geo/query/acc.cgi?acc=GSE210183).
The publicly available TCGA data used in this study are available in the cbioportal database under following study; Metastatic Melanoma (UCLA, Cell 2016, https://www.cbioportal.org/study/summary?id=mel_ucla_2016), Melanoma (MSK, NEJM 2014, https://www.cbioportal.org/study/summary?id=skcm_mskcc_2014),

Metastatic Melanoma (DFCI, Science 2015, https://www.cbioportal.org/study/summary?id=skcm_dfci_2015), Colorectal Adenocarcinoma (TCGA, Firehose Legacy, https://www.cbioportal.org/study/summary?id=coadread_tcga), Colorectal Adenocarcinoma (TCGA, PanCancer Atlas, https://www.cbioportal.org/study/summary?id=coadread_tcga_pan_can_atlas_2018), Ovarian Serous Cystadenocarcinoma (TCGA, Firehose Legacy, https://www.cbioportal.org/study/summary?id=ov_tcga) and Ovarian Serous Cystadenocarcinoma (TCGA, Nature 2011, https://www.cbioportal.org/study/summary?id=ov_tcga_pub).
The publicly available ChiPseq data used in this study are available in the ENCODE database under following accession code; (ENCFF076DCC, https://www.encodeproject.org/experiments/ENCSR000ANQ/), (ENCFF219JIE, https://www.encodeproject.org/experiments/ENCSR086FIZ/) , (ENCFF225RZA, https://www.encodeproject.org/experiments/ENCSR051VDI/) , (ENCFF063WGZ, https://www.encodeproject.org/experiments/ENCSR570YMM/) and (ENCFF042INO, https://www.encodeproject.org/experiments/ENCSR000CEL/).
All other data supporting the findings are included in this paper and its supplementary files. Source data are provided with this paper.

Human research participants

Policy information about studies involving human research participants and Sex and Gender in Research.

Reporting on sex and gender	We included gynecologic patient cohort from Kangwon National University which includes ovarian cancer, cervical cancer and non-malignant gynecological disease patients.
Population characteristics	Ovarian and cervical cancer patients and non-malignant gynecologic desease patients participated to analyse serum methionine and PBMC. They are 18 female with the age range from 30-77 (average 54.11).
Recruitment	Ovarian and cervical cancer patients and non-malignant gynecologic desease patients participated to this study.
Ethics oversight	This study was conducted according to the Declaration of Helsinki and approved by the institutional review board of Kangwon National University Hospital, with written informed consent obtained from all patients. The study protocol was approved by the Institutional Review Board (IRB) of KNUH (Chuncheon, Korea). All methods were performed in accordance with the relevant guidelines and regulations of KNUH (IRB No. KNUH-2022-03-019).

Note that full information on the approval of the study protocol must also be provided in the manuscript.

Field-specific reporting

Please select the one below that is the best fit for your research. If you are not sure, read the appropriate sections before making your selection.

☒ Life sciences ☐ Behavioural & social sciences ☐ Ecological, evolutionary & environmental sciences

For a reference copy of the document with all sections, see nature.com/documents/nr-reporting-summary-flat.pdf

Life sciences study design

All studies must disclose on these points even when the disclosure is negative.

Sample size	Sample size was determined to be adequate based on the magnitude and consistency of measurable differences between groups. The size of animal studies is between 3 to 20, which are commonly used in similar studies in literatures (Nature 585, 277–282 (2020), Mol Cancer 20, 133 (2021), Cell Metabolism 31, 250-266 (2020)).
Data exclusions	No data was excluded for all in vitro experiments. We did not perform any pre-established exclusions for in vivo experiments.
Replication	As reported in the figure legends, experiments were performed at least three times with similar results, the findings were reliably reproduced.
Randomization	For all in vivo experiments, animals were randomly assigned into a treatment group after tumor inoculation. There was no difference in the tumor burden of each experimental group at starting point of treatment. For in vitro experiment, Samples were divided into each group randomly in experiments.
Blinding	After genotyping of mice, Investigator was blinded to group allocation and data collection in in vivo experiments. Experiment and further data analysis were performed by person blinded to which animal was being assigned.

Reporting for specific materials, systems and methods

We require information from authors about some types of materials, experimental systems and methods used in many studies. Here, indicate whether each material, system or method listed is relevant to your study. If you are not sure if a list item applies to your research, read the appropriate section before selecting a response.

Materials & experimental systems

n/a	Involved in the study
☐	☒ Antibodies
☐	☒ Eukaryotic cell lines
☒	☐ Palaeontology and archaeology
☐	☒ Animals and other organisms
☒	☐ Clinical data
☒	☐ Dual use research of concern

Methods

n/a	Involved in the study
☒	☐ ChIP-seq
☐	☒ Flow cytometry
☒	☐ MRI-based neuroimaging

Antibodies

Antibodies used

FITC-conjugated anti-human CD4 antibody (1:100, Cat# 344604, RRID:AB_1937227) which was purchased from BioLegend (San Diego, CA, USA) and Alexa647-conjugated anti-human PD1 (1:100, Cat #560838, RRID:AB_2033988) antibody was purchased from BD Biosciences (Franklin Lakes, NJ, USA). Fluorescently labeled anti-CD3 (17A2), anti-CD8 (53-6.7), anti-CD4 (GK1.5, Cat# 100412 or RM4-5, Cat# 100528), anti-PD1 (RMP1-30, Cat# 109104 or 29F.1A12, Cat# 135224), LAG-3 (C9B7W, Cat# 125208), TIM-3 (B8.2C12, Cat# 134008), anti-IFN-γ (XMG1.2, Cat# 505808), anti-CD98 (RL388, Cat# 128208) antibodies from BioLegend (San Diego, CA, USA) anti-XBP1s (Q3-695, Cat# 562642) antibody from BD Biosciences (Franklin Lakes, NJ, USA)and anti-granzyme B (NGZB, Cat# 12-8898-82) antibody from eBioscience (San Diego, CA, USA)
anti-PD-1 (1:3000, D7D5W, Cat# 84651S; Cell Signaling Technology, Danvers, MA, USA, RRID:AB_2800041), anti-β-actin (1:3000, C4, Cat# sc-47778; Santa Cruz, RRID:AB_626632), anti-SLC3A2 (1:2000, Cat# MA5-29814; Invitrogen, RRID:AB_2785635), anti-SLC43A2 (1:2000, Cat# PA5-23571, Invitrogen, RRID:AB_2541071), anti-SLC7A5 (1:2000, Cat# PA5-50485, Invitrogen, RRID:AB_2635938), anti-DNMT1 (1:2000, 60B1220.1, Cat# ab13537; Abcam, RRID:AB_300438), anti-H3K4me2 (1:2000, Cat# ab32356; Abcam, RRID:AB_732924), anti-H3K4me3 (1:2000, Cat# ab8580; Abcam, RRID:AB_306649), anti-H3K9me2 (1:2000, Cat# ab1220; Abcam, RRID:AB_449854), anti-H3K27me2 (1:2000, Cat# ab24684; Abcam, RRID:AB_448222), anti-H3K79me2 (1:2000, Cat# ab177184; Abcam), anti-H3 (1:2000, Cat# ab1791; Abcam, RRID:AB_302613), anti-AMPKα1 (1:1000, Cat# 2532 L; Cell Signaling Technology, RRID:AB_330331), and anti-XBP1s (1:1000, Cat# NBP1-77681SS; Novus Biological, RRID:AB_11060050) antibodies. The membranes were incubated with HRP-conjugated anti-mouse (1:4000, ADI-SAB-100-J, Enzo, Farmingdale, NY, USA, RRID:AB_11179634) or anti-rabbit IgG antibodies (1:4000, Cat# 7074, Cell Signaling Technology, RRID:AB_2099233) for 1 h at 20 °C.

Validation

All antibodies for FACS and western blot were well-recognized clones in the field and validated by the manufacturers. These antibodies are further validated and routinely used in our lab.

Eukaryotic cell lines

Policy information about cell lines and Sex and Gender in Research

Cell line source(s)

B16F10 melanoma cells (CRL-6475TM) were purchased from the American Type Culture Collection (ATCC). MC38 colon cancer cells were purchased from the American Type Culture Collection (ATCC) in 2006. TC-1 cervical cancer cells were kindly provided by Professor Tae Woo Kim (Korea University College of Medicine, Seoul, Korea)

Authentication

B16F10 melanoma cells (CRL-6475TM) were purchased from the American Type Culture Collection (ATCC). MC38 colon cancer cells were purchased from the American Type Culture Collection (ATCC) in 2006. TC-1 cervical cancer cells were kindly provided by Professor Tae Woo Kim (Korea University College of Medicine, Seoul, Korea)

Mycoplasma contamination

The cells were tested free of mycoplasma.

Commonly misidentified lines
(See ICLAC register)

No commonly misidentified cell lines were used in the study

Animals and other research organisms

Policy information about studies involving animals; ARRIVE guidelines recommended for reporting animal research, and Sex and Gender in Research

Laboratory animals

Six-to eight-weeks old female of C57BL/6J, Rag1-/-, CD4Cre, and Prkaa1fl/fl mice were purchased from Jackson Laboratory (BarHarbor, ME, USA). Mice deficient in Prkaa1 in CD4 T cells (referred to as AMPK KO mice) and Prkaa1fl/flCD4Cre-neg (WT mice) were generated by breeding Prkaa1fl/fl with CD4Cre mice. The animals were maintained in an animal facility at 20°C to 22°C with 40% to 60% relative humidity and a 12-hour/12-hour (light/dark) cycle for at least 7 days before the experiment. All mice were fed with normal standard diet containing 14% fat, 21% protein and 65% carbohydrate (5L79; Orient Bio, Inc., Seongnam, Korea).

Wild animals

The study did not involve wild animals.

Reporting on sex

Six-to eight-weeks old female of C57BL/6J, Rag1-/-, CD4Cre, and Prkaa1fl/fl mice were used in this study.

Field-collected samples

The study did not involve samples collected from the field.

Ethics oversight

All mice experiment was approved by Institutional Animal Care and Use Committee (IACUC) of Yeungnam University (Permit number

Ethics oversight	2014-018,2022-041 and 2023-009) and Kangwon National University (KW-220727-2). Mice were housed under specific pathogen-free conditions at the animal facility of Yeungnam University and and handled according to the guidelines of the Institutional Animal Care and Use Committee (IACUC) of Yeungnam University and Kangwon National University.

Note that full information on the approval of the study protocol must also be provided in the manuscript.

Flow Cytometry

Plots

Confirm that:

☒ The axis labels state the marker and fluorochrome used (e.g. CD4-FITC).

☒ The axis scales are clearly visible. Include numbers along axes only for bottom left plot of group (a 'group' is an analysis of identical markers).

☒ All plots are contour plots with outliers or pseudocolor plots.

☒ A numerical value for number of cells or percentage (with statistics) is provided.

Methodology

Sample preparation	Tumor-infiltrating lymphocytes (TILs) were isolated by mincing tumor tissues. Minced tumor tissues were digested using an enzyme mixture containing 0.5 mg/mL of Collagenase D (Cat# 11088866001, Roche Diagnostics, Basel, Switzerland) and 0.02 mg/mL of DNase I (Cat# 10104159001, Roche Diagnostics) in RPMI 1640 medium at 37 °C for 45 min with 200rpm of continuous shaking. They were then passed through 100-µm nylon cell strainers (Cat# 352360, Falcon), and Percoll (Cat# 17-0891-01, GE Healthcare, Chicago, IL, USA) was then used to separate the TILs as previously described.<br><br>Human peripheral blood was donated by patients and centrifuged in 3 mL of Histopaque-1077 (Sigma-Aldrich, St. Louis, MO, USA) at 400 × g for 30 min. The upper supernatant plasma layer was collected, and the interphase containing PBMCs was washed thrice with phosphate-buffered saline (PBS).
Instrument	BD FACSVerse
Software	Flowjo version 10
Cell population abundance	CD4 and CD8 T cells for invitro tests were obtained from single cells of lymph nodes and spleen by using MACS Technology (Miltenyi Biotech). CD4 and CD8 T cells purity measured by FACS just after MACS sorting was more than 90 %.
Gating strategy	Details of gating strategy are represented in supplementary figure 11,12 and 13.

☒ Tick this box to confirm that a figure exemplifying the gating strategy is provided in the Supplementary Information.
